# Supplementary material for: Preoperative C-Reactive Protein-to-Albumin Ratio and Its Ability to Predict Outcomes of Pancreatic Cancer Resection: A Systematic Review
Source: Biomedicines. 2023 Jul 13;11(7):1983. doi: 10.3390/biomedicines11071983 (PMC10377035; doi:10.3390/biomedicines11071983)
Supplement: Supplementary file 1 [file biomedicines-11-01983-s001.zip › Supplementary Table S1.pdf]

Author	Representativeness of the exposed cohort	Selection of the non-exposed cohort	Ascertainment of exposure	Demonstration that outcome of interest was not present at start of study	Comparability of cohorts on the basis of the design or analysis	Assessment of outcome	Was follow-up long enough for outcomes to occur	Adequacy of follow up of cohorts	Total score
**Oshima [20]**	*	*	*	*	*	*	*	*	8
**Wijk [21]**	*	*	*	*	*	*	*	*	8
**Murakawa[22]**	*	*	*	*	*	*	*		7
**Vujic [23]**	*	*	*	*	*	*	*	*	8
**Ikuta [24]**	*	*	*	*	*	*	*	*	8
**Ikeguchi [25]**	*	*	*	*		*	*	*	7
**Wu [26]**	*	*	*	*		*	*		6
**Haruki [27]**	*	*	*	*	*	*	*	*	8

Supplementary Table S1. Methodological quality of the observational studies assessed with the Newcastle-Ottawa Scale
